# Supplementary material for: The Perception of Water Contamination and Risky Consumption in El Salvador from a Community Clinical Psychology Perspective
Source: Int J Environ Res Public Health. 2022 Jan 19;19(3):1109. doi: 10.3390/ijerph19031109 (PMC8834013; doi:10.3390/ijerph19031109)
Supplement: Supplementary file 1 [file ijerph-19-01109-s001.zip › SM - Author Group.pdf]

Affiliations of Collaborators/Memberships

“Agua Futura” Consortium

Claudia Carolina Cardoza Hernández, Karla Elisa Gómez Mejía, Thania Katerene González Nolasco, Jacqueline Lissette Flores, Lidia Esperanza Flores López, Jesica Jasmín López Villalta, Yesenia Beatriz Martínez de Guzmán, Yesenia Ivette Flores Martínez, Erika Vanessa Martínez Sánchez, Floridalma Mayorga de Ramos, Ana Catalina Mejía de Guardado, María del Carmen Merino de Lozan, Julia Susana Monge Tobar, Carolina Monserratt Jiménez de Henríquez, Laura Sofía Moreno, Keila Albertina Peña Saravia, Ingrid Carolina Pineda de Ortega, Grissel Marcela Santos Gamero, Sonia Margarita Siciliano de Serpas, Tomás Siracides Juarez Contreras, Mónica Raquel Ventura de Ramos, Wendy Yamileth Gómez Menéndez, Lennin Yasser Valle Bravo

Faculty of Medicine, University of El Salvador (UES), San Salvador, El Salvador
